# Editorial: Integrating oral health into public health: bridging gaps to reduce health disparities in the US

**DOI:** 10.3389/fpubh.2026.2001019

**Published:** 2026-10-01

**Authors:** Carla Shoff, Natalia I. Chalmers

**Affiliations:** 1 Centers for Medicare and Medicaid Services, Baltimore, MD, United States; 2 Overjet, San Mateo, CA, United States

**Keywords:** health disparities, medical-dental integration, oral health, public health, whole-person health

Oral health is inseparable from public health, yet the structures that finance, deliver, measure, and teach health care in the United States continue to treat the mouth as separate from the body. The consequences are not distributed evenly. People with low incomes, rural residents, racial and ethnic minority communities, American Indian and Alaska Native communities, people with disabilities, and those without adequate dental coverage face greater barriers to prevention and timely treatment. This Research Topic was created to move beyond describing those inequities. Its aim was to identify practical ways to integrate oral health into public health, connect medical and dental care, strengthen the workforce, use data for improvement, and demonstrate how oral health interventions can advance whole-person outcomes.

The 15 articles in this Research Topic approach that challenge from complementary levels: community partnership, workforce and interprofessional practice, care-delivery redesign, benefit policy, data science, prevention, and the oral-systemic connection (Figure 1). Together, they make one central point: integration is not a single program or referral pathway but a coordinated set of policies and practices that makes oral health visible wherever health is promoted, care is delivered, and outcomes are measured.

**Figure 1:**
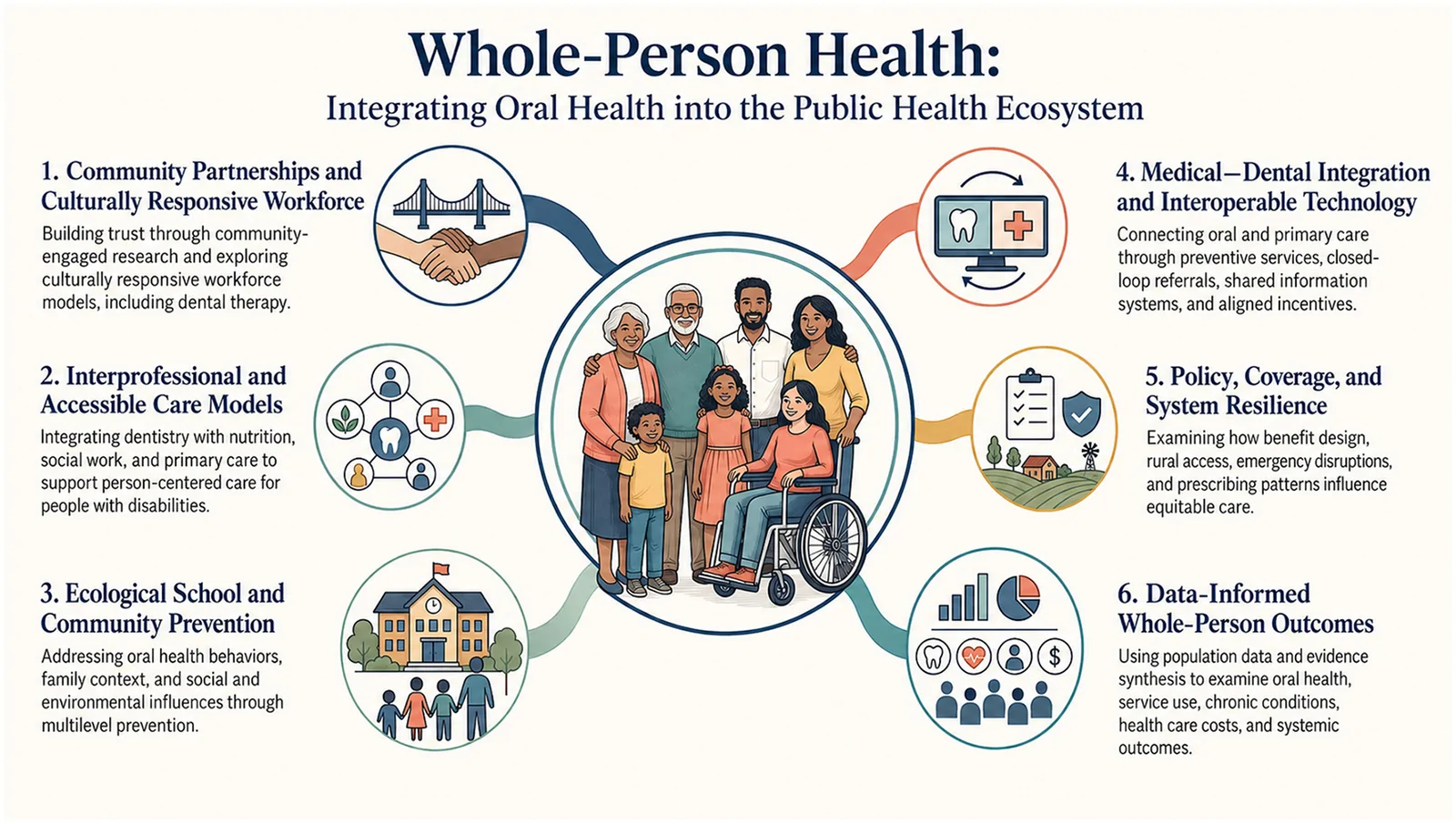
Integrating oral health into public health.

Several contributions show that equity begins with who helps define the problem and who is empowered to deliver solutions. Price et al. describe community-engaged research as a pathway for building trust, shared ownership, and sustainable action. Llaneza et al. examine provider perspectives on dental therapy and identify considerations for advocacy and implementation. A culturally responsive, community-connected workforce, they argue, could expand access, particularly for American Indian and Alaska Native communities. Gargano et al. provide an ecological model showing that school-based oral health programs are more likely to advance equity when they address multiple levels of influence rather than isolated behaviors. The common thread: workforce and program innovation must be developed with communities, not just deployed to them.

Interprofessional care is another unifying theme. Kaye et al. make the case for closer collaboration between nutrition and dentistry, given the bidirectional relationships among diet, oral disease, and chronic conditions. Morris et al. identify the distinctive contribution of social workers in dental education and care, including care coordination, attention to social drivers of health, and preparation of a more person-centered workforce. Etminan et al. describe an interdisciplinary oral-primary care model for adults with intellectual and developmental disabilities in a federally qualified health center. Longer visits, accessible equipment, trained clinicians, non-coercive approaches, and stronger referral and transportation pathways were central to a better patient experience. These articles show that integration requires changes in team composition, training, workflows, accessibility, and organizational culture.

The MORE Care initiative reported by Vermillion et al. illustrates how those principles can be implemented across multiple practices and communities. In an Ohio pilot involving nearly 20,000 children across six counties, medical practices incorporated oral assessments, fluoride varnish, and referrals, supported by performance-based payment. Increased preventive services and referrals indicate that medical-dental integration can extend beyond safety-net settings. The reported health-information-technology and data-sharing barriers are equally instructive: durable integration depends on interoperable systems, closed-loop referrals, aligned incentives, and measures that hold organizations accountable for completed care rather than attempted handoffs.

The Research Topic also documents how emergencies expose fragile systems and can accelerate change within them. Meyer et al. found increased use of silver diamine fluoride among Medicaid-enrolled children during the COVID-19 public health emergency, which points to the value of minimally invasive, non-aerosolizing care when conventional delivery is disrupted. Burns et al. found that endodontic utilization declined less during the emergency and increased afterward, while more comprehensive Medicaid dental benefits were associated with greater utilization. Their results place benefit design at the center of resilience and equity. Shoff et al. identify rural-urban, racial and ethnic, and community-level socioeconomic differences in dental opioid prescribing among Medicaid beneficiaries; policy responses therefore need to be stratified and monitored for unintended inequities. Prevention must also extend beyond a procedure: Buscemi et al. present the protocol for the PROTECT randomized trial, which will evaluate a community-health-worker-delivered intervention designed to reduce recurrence after pediatric dental surgery by addressing toothbrushing, sugar consumption, parenting practices, and family context.

Data can help public health systems anticipate needs, but it must be used carefully. Zanwar et al. demonstrate the use of multiple artificial-intelligence models with nationally representative survey data to identify features associated with dental service use during the pandemic. This work offers a foundation for population surveillance and targeting. Future applications should emphasize transparent validation, evaluation of potential bias, and a clear distinction between prediction and causation. It is not enough for data-driven integration to identify who is least likely to receive care; that finding should trigger accountable action to remove the barriers producing the pattern.

Finally, three articles situate oral health within whole-person outcomes. Santoro et al. report an association between periodontal treatment and lower medical spending among commercially insured adults with cardiovascular disease; because the study is observational, the finding should motivate prospective evaluation rather than causal claims. Sha et al. synthesize and organize evidence for non-surgical periodontal management among people with type 2 diabetes across assessment, risk-factor control, oral hygiene, diet, mechanical treatment, adjunctive measures, and follow-up. Song et al.'s umbrella review links tooth loss with multiple adverse health outcomes, but rates the overall evidence predominantly low or very low, with heterogeneity and potential bias. This balance, recognizing consistent signals without overstating certainty, is essential to credible integration.

Across the Research Topic, the direction is clear. Oral health must be embedded in public health surveillance, primary care, chronic-disease management, nutrition, social care, emergency preparedness, payment, and workforce policy. The next phase should test implementation at scale; measure access, experience, clinical outcomes, costs, and equity together; and report who benefits, who is missed, and why. Integration will be successful when oral health is no longer an optional add-on, but a routine and accountable component of whole-person and population health.

